# Supplementary material for: The interactome of intact mitochondria by cross-linking mass spectrometry provides evidence for coexisting respiratory supercomplexes
Source: Mol Cell Proteomics. 2017 Dec 8;17(2):216–32. doi: 10.1074/mcp.RA117.000470 (PMC5795388; doi:10.1074/mcp.RA117.000470)
Supplement: Supplemental Data [file supp_RA117.000470_133922_0_supp_23978_fzffwf.zip › spectra_annotation/mito_DR_spectra_annotation/470-1-4-1-11-1.pdf]

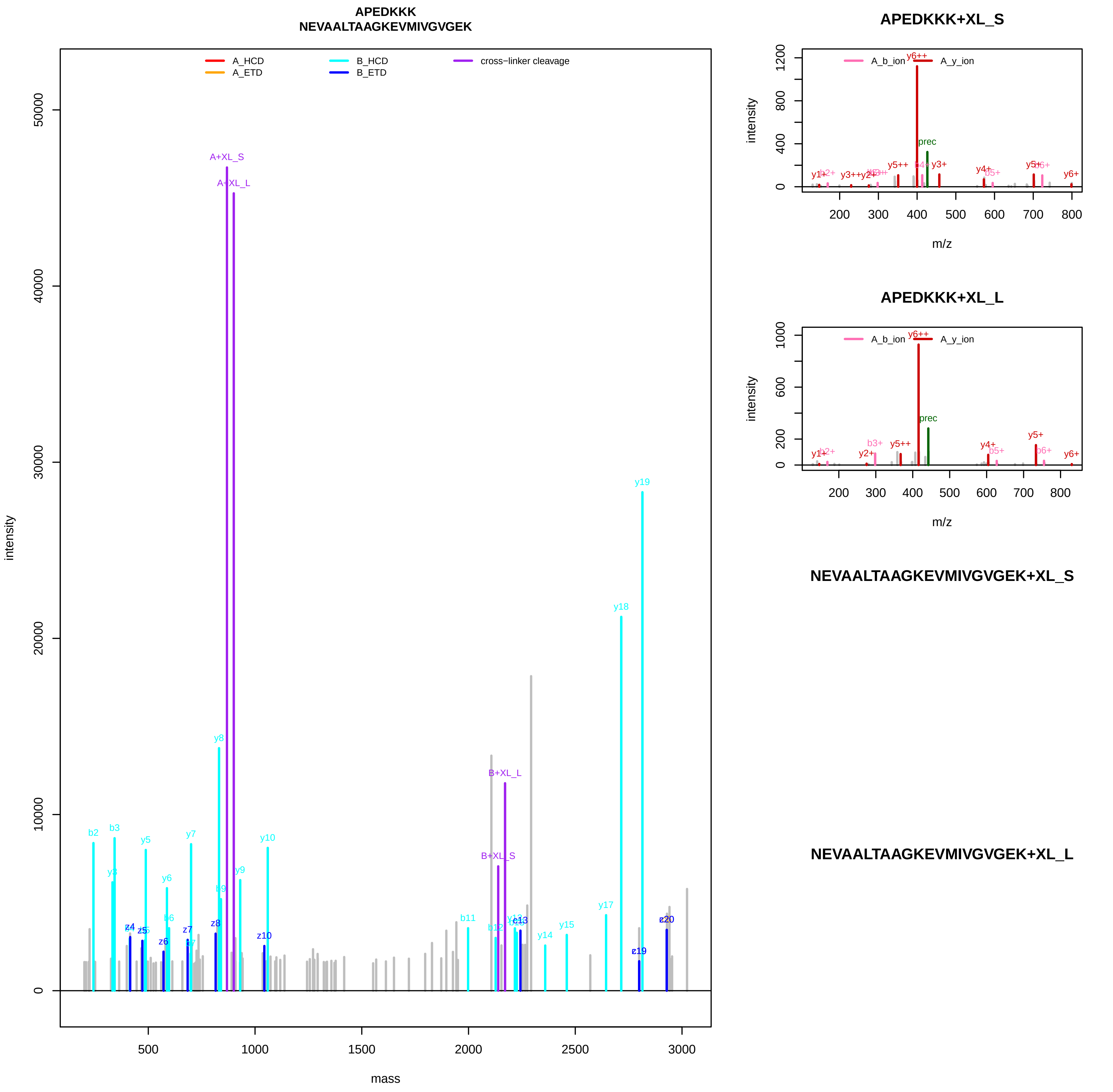

APEDKKK
NEVAALTAAGKEVMIVGVGEK
A_HCD
A_ETD
B_HCD
B_ETD
cross−linker cleavage
A+XL_S
A+XL_L
B+XL_L
B+XL_S
y19
y18
y17
y15
y14
y10
y9
y8
y7
y6
y5
b2
b3
b11
b12
z4
z5
z6
z7
z8
z10
z19
z20
c13
intensity
mass
APEDKKK+XL_S
APEDKKK+XL_L
A_b_ion
A_y_ion
y6++
prec
y5++
y3+
y4+
y5+
y6+
y1+
b2+
b3+
b5+
b6+
y2+
m/z
NEVAALTAAGKEVMIVGVGEK+XL_S
NEVAALTAAGKEVMIVGVGEK+XL_L
